# HER2/CEP17 Ratios and Clinical Outcome in HER2-Positive Early Breast Cancer Undergoing Trastuzumab-Containing Therapy

**DOI:** 10.1371/journal.pone.0159176

**Published:** 2016-07-27

**Authors:** Albina Stocker, Marie-Luise Hilbers, Claire Gauthier, Josias Grogg, Gerd A. Kullak-Ublick, Burkhardt Seifert, Zsuzsanna Varga, Andreas Trojan

**Affiliations:** 1 Breast-Center Zürich, Zürich, Switzerland; 2 Department of Clinical Pharmacology and Toxicology, University Hospital Zurich, Zürich, Switzerland; 3 Department of Epidemiology, Biostatistics and Prevention Institute, University of Zurich, Zürich, Switzerland; 4 Institute of Surgical Pathology, University Hospital Zurich, Zürich, Switzerland; University of Torino, ITALY

## Abstract

**Background:**

Adjuvant therapy comprising the HER2 receptor antagonist trastuzumab is associated with a significant improvement in disease-free and overall survival as compared to chemotherapy alone in localized HER2-positive breast cancer (BC). However, a subset of HER2-positive tumors seems to respond less favorably to trastuzumab. Various mechanisms have been proposed for trastuzumab resistance, such as high *HER2* to *Chromosome 17* FISH (*HER2/CEP17*) ratios and the possibility that single agent trastuzumab may not suffice to efficiently block HER2 downstream signaling thresholds. In a retrospective analysis we evaluated whether *HER2/CEP17* ratios might have an impact on disease-free survival (DFS).

**Methods:**

Clinical records of Stage I-III BC patients with HER2-positive tumors were reviewed at our institution from 2007–2013. We analyzed demographics, tumor characteristics including tumor size and grade, lymph node involvement and estrogen receptor expression as well as treatment with respect to chemotherapeutic regimens from the clinical charts. *HER2/CEP17* ratios were determined by routine pathology analysis using in situ fluorescent hybridization (FISH). Upon statistical preview we defined three groups of *HER2* amplification based on FISH ratio (2.2 to 4, >4 to 8, >8), in order to evaluate an association between *HER2* gene amplification and DFS with trastuzumab containing therapies. DFS was analyzed using Cox-regression.

**Results:**

A total of 332 patients with HER2-positive BC were reviewed. Median age was 54 (range 23–89) years. The majority of tumors were classified T1 (50%) or T2 (39%), node negative (52%) and of high grade G3 histology (70%). We identified 312 (94%) tumors as immunohistochemistry (IHC) score 3+ and *HER2/CEP17* ratios were available from 278 patients (84%). 30% (N = 84) had tumors with high *HER2/CEP17* ratios (>8). Univariate analysis found no correlation between outcome, age, histological grade, sequence as well as anthracycline content of chemotherapy. However, a prognostic impact was detected for tumor size (p = 0.02), nodal status (p<0.01), proliferation index (p<0.01), level (≥20%) of estrogen receptor expression (p = 0.03) and neoadjuvant therapeutic setting (p = 0.03), respectively. Importantly, univariate and multivariable analysis revealed that standard trastuzumab containing chemotherapy resulted in impaired disease free survival among tumors with FISH ratio >8 (p<0.01). Although less pronounced, a similar association was found also with respect to high *HER2* gene copy numbers (>12) and DFS (p = 0.01).

**Conclusions:**

In early BC patients, tumors with high *HER2* amplification ratios (>8), may less likely respond to standard trastuzumab-containing therapies. Although, we obtained a similar effect for high *HER2* gene copy numbers, this provides only an indirect speculation and not a proof that high *HER2/CEP17 ratios* may induce HER2 resistance.

## Introduction

The *HER2* oncogene is located on chromosome 17, and is amplified in approximately 20% of early stage breast cancers thus resulting in overexpression of transmembrane receptor tyrosine kinase HER2. HER2 activation may occur through heterodimerization with other ligand-bound receptors, by spontaneous homodimerization of truncated receptor isoforms or of full-length HER2 when expressed at high density, and by metalloproteinase-mediated cleavage of the extracellular domain [1], [2], [3]. *HER2* amplification and consecutive receptor overexpression are negative predictors of both disease-free survival (DFS) as well as overall survival (OS) in patients with BC and are associated with rapid disease progression, resistance to endocrine therapies as well as higher metastatic potential [4], [5]. The humanized monoclonal antibody trastuzumab binds to the extracellular domain of Her-2/neu thereby counteracting mitogenic and survival signaling by the kinase receptor thus conferring antibody-dependent cellular cytotoxicity [6]. To date, the selection of BC types likely to respond to trastuzumab, is based on the detection of sufficient Her-2 protein on the cell surface by immunohistochemistry (IHC) and in situ hybridization techniques as FISH, SISH or CISH which identifies the number of *HER2* gene copies on chromosome 17 also in relation to centromere 17 (*CEP17*) copies per nucleus. Until 2013, most classification systems including the ASCO/CAP guidelines, consider tumors as amplified if the *HER2/CEP17* ratio (R) exceeds 2.2 or, in the absence of CEP17 assessments, if the absolute *HER2* copy number (CN) exceeds 6 [7], [8]. Since the adoption of adjuvant trastuzumab as a standard component of therapy for HER-2/neu-positive early-stage as well as advanced BC about 10 years ago, outcomes of many patients have substantially improved with respect to recurrence and death [6], [9]. Based on results from randomized clinical trials, a trastuzumab-containing regimen for up to 1 year is now considered standard of care for patients with HER2-positive tumors larger than 0.5 and 1 cm in size, respectively [10]. However, subsets of HER2-positive early stage as well as metastatic BC do not seem to likewise respond to trastuzumab [4], [11]. A variety of mechanisms have been proposed for a presumed trastuzumab resistance such as high levels of *HER2* gene amplification resulting in insufficient blockage of HER2 downstream signaling thresholds [11], [12], [13], [14]. While the benefit of anti-HER2 directed therapy has been explored in various neoadjuvant and adjuvant settings [15], [16], [17], prospective clinical trials for BC patients with high *HER2* gene amplification treated with trastuzumab, have not been conducted so far. In a retrospective analysis we evaluate whether the HER2 status, based on *HER2/CEP17* ratios as well as *HER2* copy numbers would predict for the response to trastuzumab in early BC patients.

## Patients and Methods

The study was a part of a retrospective larger breast cancer project, which was previously approved by the Ethical Commission of the Canton Zürich (KEK-ZH-2012-553). Informed written or oral consent has been obtained from the participants. In addition, data were analyzed anonymously. Clinical records of all women with Stage I-III HER2-positiveBC who were treated at the Breast Center Zürich between 2007 and 2013 were reviewed and clinical characteristics as well as pathological reports were categorized and made available for further analysis. Patients received either neoadjuvant or adjuvant standard chemotherapies containing trastuzumab. Data on demographics, tumor size (T), nodal status (N), histological grade (G), *HER2* amplification levels, and clinical outcomes were analyzed. A systematic review was performed, in order to evaluate the association between *HER2* to *chromosome 17* FISH ratios (*HER2/CEP17* ratio; R) and clinical outcome. In all patients eligible, HER2 positivity was determined by IHC and fluorescence in-situ hybridization (FISH). HER2 status along with the processing and reporting of the breast biopsies/specimens was carried out in the Institute of Surgical Pathology at the University Hospital Zurich, Switzerland. HER2 status was routinely assessed primarily on the preoperative core/vacuum biopsies. In case of insufficient tumor tissue on the biopsies, lack of biopsy or of equivocal test results on the biopsies, HER2 status was assessed on the consecutive surgical specimen. Analytical procedures of the HER2 assays including antibodies, technical modalities and hybridization probes (FISH) were described in detail elsewhere [18], [19]. Scoring of HER2 status was performed following the at the time current FDA and ASCO/CAP guidelines, as described and summarized in previous work [18], [20], [21], [22]. Due to our observational period, practically all patients were evaluated for HER2 expression according to the 2007 ASCO/CAP guidelines [20]: Equivocal HER2 IHC was scored 2+ if strong complete intense membrane staining occurred in <30% of the invasive tumor cells or weak to moderate heterogeneous complete staining in > 10% of the invasive tumor cells, while a positive IHC score 3+ was determined if a strong complete homogenous membrane staining occurred in >30% of the analyzed invasive tumor cells. FISH *HER2* positivity was based on the *HER2/CEP17* ratio (> 2.2) and on the average gene copy numbers. Gene copy numbers of >6 and cluster formation (6 copies by smaller clusters and 12 copies by larger clusters) were also defined as positive status. HER2 equivocal status by FISH was defined as a *HER2/CEP17* ratio of 1.8–2.2.

### Statistical methods

Disease free survival (DFS) was the primary end point, defined as local, regional, or distant recurrence of BC, contralateral BC (except for lobular carcinoma in situ of the breast). The duration of DFS was defined as the time from diagnosis to the first event. The date of recurrence was coded as the first clinical and/or pathological confirmed incidence of new or recurrent invasive BC. DFS was estimated by the Kaplan-Meier method and analyzed using the log-rank test and Cox regression. All statistical analyses were performed with the statistical software IBM SPSS Statistics (Version 22.0. Armonk, NY: IBM Corp, USA). Hazard ratios (HR) were computed using Cox regression and presented with 95% confidence interval (CI). Two-sided p values p<0.05 are considered statistically significant.

## Results

A total of 332 patients with HER2-positiveBC were reviewed within the observational period. Median follow up was 45 (mean 49) months. Relapse occurred in a total of 55/323 patients (17%), of which 14 (4%) had died during the observational period. OS and DFS of all patients after at 7 years was 92.8% (±2.1% SE) and 70.6% (±4.1% SE), respectively. Median age was 54 (range 23–89) years. Univariate analysis was performed for all available clinical parameters (Table 1).

**Table 1:** Univariate analysis of clinicopathological data and DFS.

	N (percentage)	P value	HR (95%CI)
**Age** (median, range)	54 (23–89)	0.05	1.02 (CI 1.00–1.04)
**Tumor stage** (n = 313)			
pT1/2	279 (89%)	0.02	2.2 (CI 1.1–4.3)
pT3/4	24 (11%)		
**Nodal status** (n = 328)			
pN0/1	268 (82%)	<0.01	2.7 (CI 1.6–4.7)
pN2/3	60 (18%)		
**Histological grade** (n = 322)			
G1/2	96 (30%)	0.13	1.7 (CI 0.86–3.2)
G3	226 (70%)		
**Proliferation (Ki-67)**			
≥30%	114 (54%)	<0.01	2.9 (CI 1.3–6.5)
**Hormone receptors** (n = 328)			
ER+	209 (64%)		
ER>20%	23 (7%)	0.03	0.54 (CI 0.32–0.93)
PR neg	162 (49%)		
**Chemotherapy** (n = 309)			
Neoadjuvant	46 (15%)	0.03	1.9 (CI 1.05–3.5)
Adjuvant	263 (85%)		
**Chemotherapy** (n = 316)			
Anthracyclines	181 (57%)	0.11	0.65 (CI 0.38–1.11)
non Anthracyclines	135 (43%)		
***HER2/CEP17* Ratio terziles** (n = 278)			
2.2–4	94 (34%)		1.0
>4–8	100 (36%)	<0.01	2.1 (CI 0.8–5.5)
>8	84 (30%)		4.6 (CI 1.9–11.3)
***HER2 copy number* terziles** (n = 278)			
≤12	100 (36%)		1.0
13–20	97 (35%)	0.01	3.4 (1.5–7.5)
>20	81 (29%)		2.2 (0.95–5.2)

**Fig 1:**
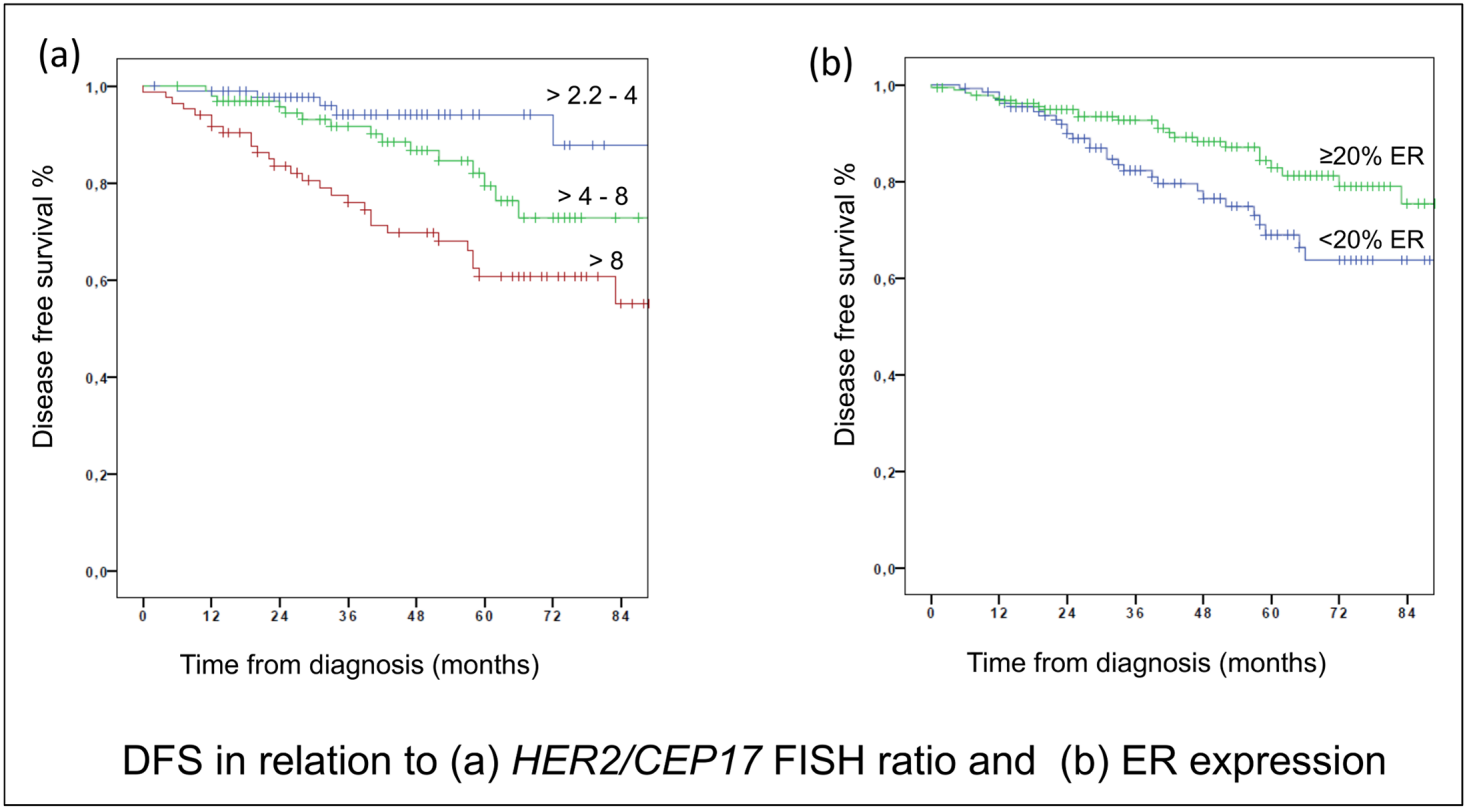
DFS in relation to (a) *HER2/CEP17* FISH ratio and (b) ER expression.

Age did not reveal a clear effect on DFS (p = 0.05) also with respect to patients of age younger than 45 years (p = 0.99). The majority of tumors were classified T1 or T2 (50% and 39%, respectively), and tumor size did significantly affect prognosis also when we looked at tumor categories T1-T2 and the less frequent and larger T3-T4 tumors (11%) (p = 0.02). About half of the patients were diagnosed with nodal negative disease (52%). As was expected nodal status also had a significant impact on prognosis, in particular when more than 4 lymph nodes (18%) were involved (p<0.01). Two thirds (70%) of all tumors were of high histological grade G3. However, surprisingly histological grading did not apparently affect DFS when G1 (1%) and G2 tumors (29%) were compared to the prevalent G3 tumors, respectively. Median proliferation index (as Ki-67 labelling index) was calculated as 30% (range 5–90%) and clearly correlated with impaired prognosis (p<0.01) according to the univariate analysis. From the 209 tumors that stained positive for estrogen receptors (ER) only 18 (9%) exhibited an expression of less than 20% ER, which also had prognostic relevance (p = 0.03). This finding might indicate that a certain amount of ER in the carcinoma cells is mandatory for adjuvant antihormonal efficacy also in HER2-positive tumors [23]. No effect could be determined for tumors that stained positive for PR (51%) in our cohort. Median duration of anti-hormonal treatment reported was 44 months, thus indicating a reasonable adherence for the interpretation of our results. According to clinical records, 85% of the patients received adjuvant antihormonal therapy, of which again 25% consisted of tamoxifen, 30% of aromatase inhibitor whereas 6% were treated by documented switch, respectively. Supposedly, prognosis seemed to be related to neoadjuvant systemic regimens when compared to the more commonly applied adjuvant therapies (p = 0.03), a finding that may be due to the fact that neoadjuvant approaches are frequently performed in more advanced stages. However, seven patients (2%) completed 1 year of 3 weekly trastuzumab but did not continue adjuvant chemotherapy after their first infusion. With respect to chemotherapies, half of the patients (51%) received 4 cycles of the anthracycline containing regimens epirubicine and cyclophosphamide followed by twelve weekly cycles of paclitaxel and concomitant trastuzumab, less frequently three cycles of FEC100 (5-FU, epirubicine, cyclophosphamide) followed by three cycles docetaxel (8%). Alternatively, four to six cycles of adjuvant docetaxel and cyclophosphamide (16%), or six cycles of docetaxel and carboplatin (9%) were administered concomitantly to trastuzumab. Neoadjuvant chemotherapy was preferentially applied according to the NOAH regimen in 15% of the patients. However, no significant impact on DFS, neither by any of the therapeutic regimens nor by use of anthracyclines as component of systemic therapy could be detected (p = 0.11). IHC score 3+ was identified in 312 (94%) tumors and *HER2/CEP17* ratios were available from 278 patients (84%). According to previous reports, we assumed that 15–30% of the tumors will exhibit high *HER2* levels, as defined by FISH ratio >8.0. We assessed ratios according to clinico-pathological convertible terziles (>2.2 to 4; >4 to 8; >8) and conducted univariate statistical analysis. Patients, whose tumors revealed *HER2/CEP17* ratios >8 (n = 52) had a significantly increased risk for disease specific relapse (p<0.01) (Fig 1a). Of note, the negative effect of a high *HER2/CEP17* ratio seemed in part alleviated in patients with a significant ≥20% ER positivity as compared to patients with low ER expression (p = 0.03) (Fig 1b). Also, a multivariate analysis was performed by backward stepwise variable selection and results are presented in Table 2. Accordingly, *HER2/CEP17* ratios >8 (p<0.01) as well as tumor size (p = 0.03) and nodal status (p<0.01) remained significant negative prognostic factors.

**Table 2:** Multivariate analysis of clinicopathological data and DFS after backward stepwise variable selection.

	P value	HR (95%CI)
***HER2/CEP17* Ratio terziles**		
2.2–4		1.0
>4–8	<0.01	2.1 (CI 0.8–5.8)
>8		4.6 (CI 1.8–11.7)
**Tumorstage**		
pT1/2	0.03	2.3 (CI 1.1–4.6)
pT3/4		
**Nodal status**		
pN0/1	<0.01	2.8 (CI1.5–5.3)
pN2/3		

Since other authors suggest a possible correlation between *HER2* copy numbers and outcome, in addition, we also made available gene copy number terziles (≤12; 12–20, >20) for an appropriate univariate statistical analysis (Table 1). Although less pronounced, patients whose tumors revealed high *HER2* copy numbers (>12) exhibited an impaired disease free survival (p = 0.01).

## Discussion

Previous work suggests that single agent use of the HER2 receptor antagonist trastuzumab might be less efficient in blocking HER2 downstream signaling thresholds in the presence of high level of *HER2* gene amplification or high *HER2/CEP17* ratios [14], [24]. Here, we demonstrate a significantly shorter DFS in early stage *HER2* FISH positive BC patients treated with trastuzumab containing chemotherapy if *HER2/CEP17* ratios in the tumors were >8. However, the prognostic impact of high ratios appeared to be less prominent if tumors also exhibited >20% estrogen receptor expression. In order to strengthen our hypothesis in this larger series of patients *HER2* gene copy numbers were assessed for correlation with clinical outcome. Although less pronounced, high *HER2* copy numbers also indicated an impaired DFS. Regarding resistance to trastuzumab, in metastatic settings Lee and coworkers [25] reported in a larger cohort of HER2-positive primary BC that co-overexpression of the epidermal growth factor receptor (EGFR 1) may also be indicative for a poor prognosis and is associated with hormone receptor negativity. Others have demonstrated that co-overexpression of HER2/HER3 may significantly impair OS [26], indicating that ligand dependent dimerization of HER2-HER3 might reduce intracellular signal transduction such as PI3K/Akt. A recent analysis in 127 patients with metastatic HER2 positive BC receiving trastuzumab-based treatment revealed a significantly shorter time to first metastasis in tumors exhibiting low- and high-level *ERBB2/ CEP17* ratios [27], an outcome that seemed more prominent when absolute *ERBB2* copy numbers were >13. In this study, however, *HER2/CEP17* ratios, turned out to be an independent predictor of complete and partial response upon HER2 directed treatment, whereas the absolute *HER2* copy number did not. The later finding might indicate altered genetic patterns in metastatic BC, and differs from our observation in early BC that *HER2/CEP17* ratios *and HER2* copy numbers are associated with response to trastuzumab. Others, however, have reported that an advanced clinical stage may as well predict for high *HER2* amplification levels but seems to frequently lack correlation towards HER2 ratios [28].

In a rather small dataset of metastatic BC patients Bates and coworkers [29] also identified a subpopulation with very high HER2 protein expression levels associated with resistance to trastuzumab. In addition, high HER2 protein expression associated with a continuous increase of FISH *HER2/CEP17* ratios as well as copy numbers. Using tissue microarray analysis [30], more recently, a larger number of metastatic and primary BC was analyzed for HER2 status. Although there was a significant correlation between HER2 status determined by IHC and by FISH, only *HER2* gene amplification status correlated with outcome, thereby indicating greater utility for FISH in routine clinical settings.

In an adjuvant chemotherapeutic setting, Perez and coworkers [31] found that continuous *HER2* mRNA expression level measured by RT-PCR was not significantly associated with the magnitude of benefit from the addition of trastuzumab. Of note, also in this study no association was observed for HER2 IHC and *HER2/CEP17* FISH ratio. Previously, a sub-analysis of the N9831 trial suggested that patients with resected HER2-positive BC, treated with trastuzumab containing chemotherapy may exhibit an impaired prognosis if FISH *HER2/CEP17* ratios were ≥15 [32]. However, no linear dose-effect between the level of *HER2* gene amplification and response to trastuzumab was observed and no difference was found between normal [disomic] and polysomic chromosome 17, respectively, a finding also described in previous work [7].

The retrospective analysis of SWOGS9313/Int0137 trial in 303 early-stage BC patients [11] found that OS and DFS rates were strongly and adversely associated with *HER2/CEP17* ratios of greater than 4.0. High-level *HER2* amplification also remained a prognostic marker upon adjustment for menopausal status, hormone receptors, treatment, nodal status and tumor size. Albeit not statistically significant, a subgroup analysis conducted by the HERA investigators suggested a trend towards decreasing effectiveness of trastuzumab with increasing FISH ratios of greater than 8.0, which became less evident when *HER2* was quantified by copy numbers only [7]. A similar observation, however, was made upon data analysis in our patient cohort. Of note, the subset of patients with very high *HER2* gene copy number identified in HERA contained 494 patients, rendering it unlikely that the observation was purely an artifact of sample size. Thus, several lines of evidence, including those from larger well-designed clinical trials of trastuzumab in the adjuvant setting, indicate that a HER2 driven resistance to trastuzumab can occur. So far, a number of hypotheses have been proposed to explain why some patients fail to achieve optimal outcomes on trastuzumab, including activation of the PI3 kinase signaling pathway as a result of HER2–HER3 heterodimerisation, as well as constitutive activation of downstream signaling mediated by a truncated form of the HER2 receptor, p95/HER2 [27], [33], [34].

To date, only few studies indicate that high *HER2* copy numbers (>12) may be even associated with a consistently better response compared to patients with intermediate *HER2* copy numbers (6–12) [35]. Nevertheless, conclusions were based on a smaller subset of patients being score2+ by IHC, and thus did not reach statistical power. Of note, we found a similar association between high and intermediate *HER2/CEP17* ratios by analyzing quintiles (data not shown). While a HERA trial sub-analysis, found an inverse correlation between FISH ratio and ER-positivity, of note, results from the N9831 trial demonstrated a lower rate of relapse in ER- positive BC than in hormone receptor-negative tumors [32]. The later finding was also a prognostic indicator in our present analysis if ER expression exceeded 20%. In a neoadjuvant and anthracycline-free setting, however, cancers with >10 *HER2* gene copies per cell were reported to achieve significantly higher pCR rates, although this did not translate into a DFS or OS benefit [36]. For now, highly reliable and clinically meaningful treatments are reported in prospective trials when combining dual anti Her2 blockade with either pertuzumab or lapatinib [17], while other efforts, such as combined HER2 and PI3K/Akt/mTOR blockade, to overcome resistance towards single agent trastuzumab, tend to be explored with more caution [37].

Our study has several limitations, such as retrospective character and the fact that from a limited number of neoadjuvant settings no conclusions upon the use of single agent trastuzumab and pathological response rate or risk of recurrence can be drawn. We also did not explore the possible correlation of DFS with high HER2 protein expression or an associated expression of receptor fragments that in turn may affect the binding capacity of trastuzumab [38]. Taken into account complex genetic patterns in BC that arise from aneusomy of chromosome 17 (including polysomy and monosomy), co-localization of the *CEP17* and the *HER2* signals as well as intratumoral genetic heterogeneity and supposedly discordant FISH results [39], [40], [41], [42], our results provide only an indirect speculation and not a proof that high *HER2*/CEP17 ratios contribute to trastuzumab resistance.
